# The Effect of World Trade Center Exposure on the Timing of Diagnoses of Obstructive Airway Disease, Chronic Rhinosinusitis, and Gastroesophageal Reflux Disease

**DOI:** 10.3389/fpubh.2017.00002

**Published:** 2017-02-08

**Authors:** Xiaoxue Liu, Jennifer Yip, Rachel Zeig-Owens, Jessica Weakley, Mayris P. Webber, Theresa M. Schwartz, David J. Prezant, Michael D. Weiden, Charles B. Hall

**Affiliations:** ^1^Department of Medicine, Montefiore Medical Center, Bronx, NY, USA; ^2^Bureau of Health Services, Fire Department of the City of New York, Brooklyn, NY, USA; ^3^Department of Epidemiology and Population Health, Albert Einstein College of Medicine, Bronx, NY, USA; ^4^Department of Medicine, New York University School of Medicine, New York, NY, USA

**Keywords:** 9/11, firefighters, piecewise survival model, aerodigestive, obstructive airways disease

## Abstract

**Objectives:**

In a cohort of rescue/recovery workers exposed to the dust that resulted from the collapse of the World Trade Center (WTC), we assessed how a diagnosis of obstructive airways disease (OAD) affected the likelihood of a subsequent diagnosis of chronic rhinosinusitis (CRS) or gastroesophageal reflux disease (GERD). We also assessed whether OAD acted as a mediator of the association between exposure to the WTC rescue/recovery effort and CRS and GERD diagnoses.

**Methods:**

In this prospective cohort study, we analyzed Fire Department of the City of New York physician diagnoses of OAD, CRS, and GERD that were first documented between September 11, 2001, and September 10, 2011, among 8,968 WTC-exposed firefighters. We used piecewise exponential survival models to evaluate whether OAD was a risk factor for either CRS or GERD and to assess OAD as a possible mediator.

**Results:**

An OAD diagnosis significantly increased the risks for subsequent CRS [relative rate (RR), 4.24; 95% CI, 3.78–4.76] and GERD (RR, 3.21; 95% CI, 2.93–3.52) diagnoses. Further, 21% of the WTC exposure effect (high vs. low intensity) on GERD and 13% of the effect (high vs. low intensity) on CRS were mediated by a prior OAD diagnosis.

**Conclusion:**

Individuals with an OAD diagnosis had elevated risks for subsequent diagnoses of CRS or GERD. Part of the effect of WTC exposure on CRS and GERD diagnoses is mediated by prior diagnoses of OAD; this mediation effect of OAD may reflect biological pathways or healthcare utilization practices.

## Introduction

The destruction of the World Trade Center (WTC) buildings in New York City after the terrorist attack on September 11, 2001 resulted in a massive dust cloud containing partially combusted and/or pulverized wood, paper, and jet fuel; pulverized construction materials including asbestos, glass, silica, fiberglass, and concrete; complex organic chemicals; lead and other metals; and other potentially hazardous materials (1). Adverse respiratory effects of WTC exposure have been widely documented and have shown consistent dose–response relationships (2, 3). We and others have found a high postexposure health burden of aerodigestive conditions, a category that includes lower respiratory diseases such as asthma, chronic bronchitis, and COPD/emphysema, which together are categorized as obstructive airways disease (OAD); upper respiratory diseases that were predominantly chronic rhinosinusitis (CRS); and gastroesophageal reflux disease (GERD) (2, 4, 5). In the decade and a half since the disaster, a subgroup of WTC-exposed workers have experienced chronic inflammation at mucosal surfaces in the nose, sinuses, and lungs, producing CRS (3, 6), reactive airway disease, and GERD, which may be due to caustic esophageal exposure in the context of accidental ingestion. The common pathway for these conditions may be postexposure airway inflammation and hyperresponsiveness (7). By 2015, nearly 30% of Fire Department of the City of New York (FDNY) WTC rescue/recovery workers had a physician diagnosis of CRS, 28% GERD, and 24% OAD (8).

High comorbidity rates of WTC-related aerodigestive conditions have been commonly reported (9–12). Notably, the percentage of WTC rescue/recovery workers diagnosed with all three aerodigestive conditions ranged from approximately 10–30% (8, 10, 11). Clinical reasons for disease co-occurrence remain unknown. GERD may cause lower and upper respiratory diseases or exacerbate persistent airway irritation (13, 14). Alternatively, OAD or CRS may cause or exacerbate GERD through mechanically induced inflammation (e.g., cough, postnasal drip, mucous), drug effects (theophylline, corticosteroids), or shared neurological pathways.

We have previously reported that FDNY WTC rescue/recovery workers were more frequently diagnosed with OAD than CRS in the months and years after exposure, but that over time, diagnoses of CRS increased (2, 3, 15). Similarly, about 6 years postexposure, GERD diagnoses, which had the lowest immediate post-9/11 incidence of these aerodigestive conditions, started to increase (unpublished data). The role, if any, of an early OAD diagnosis in subsequent diagnoses of CRS and/or GERD was unclear. Given our access to FDNY’s electronic medical records system, which includes dates of diagnoses, we explored the associations between post-WTC exposure OAD diagnoses in relation to CRS and GERD in a cohort of FDNY WTC-exposed firefighters. Specifically, our aims were to 1) assess how a diagnosis of OAD affects the likelihood of a subsequent diagnosis of CRS or GERD and 2) assess whether OAD acts as a mediator of the associations between WTC exposure and CRS and WTC exposure and GERD.

## Materials and Methods

The source population consisted of FDNY male firefighters who were active (i.e., non-retired) on September 11, 2001 and who arrived at the WTC site to participate in the rescue/recovery effort on or before September 24, 2001; who gave informed consent for research; and who had at least one visit to the FDNY Bureau of Health Services (FDNY-BHS) for treatment of any medical condition after exposure (*N* = 10,181). After excluding firefighters with pre-WTC exposure evidence of OAD, CRS, or GERD in their FDNY-BHS medical records, the final analysis cohort consisted of 8,968 participants.

Demographic information such as age, race, and retirement status was obtained from the FDNY employee database. WTC exposure intensity was obtained from participants’ first post-9/11 health questionnaire. Since 1996, FDNY-BHS has used an electronic medical record system, which contains in-house physician diagnoses and information from diagnostic tests such as endoscopy, spirometry, methacholine challenge tests, and chest CT scans.

WTC exposure intensity was categorized by time of initial arrival to the WTC site: on the morning of September 11, 2001 (high); in the afternoon of September 11, 2001, or anytime on September 12, 2001 (moderate); and on any day between September 13, 2001, and September 24, 2001 (low) (2, 3, 15).

New-onset OAD, CRS, and GERD conditions were diagnosed by FDNY-BHS physicians. We reviewed the FDNY electronic medical record database for the first documented mention of these conditions between September 11, 2001, and September 10, 2011. We used the same case definitions for OAD and CRS as previously described (2, 3, 15). Briefly, an OAD case had any of the following: 1) two or more diagnoses of asthma documented at least 30 days apart, 2) two or more diagnoses of COPD/emphysema documented at least 30 days apart, or 3) two chronic bronchitis diagnoses recorded within 1 year of each other, followed by at least one additional chronic bronchitis diagnosis within the next 3 years. A CRS case required a diagnosis of either CRS or irritant chronic rhinitis and at least one abnormal diagnostic test result (either nasal laryngoscopy or sinus CT scan). A GERD case required two or more diagnoses documented at least 30 days apart of gastroesophagitis, esophagitis reflux, or Barrett’s esophagus.

### Statistical Analysis

We separately fit piecewise exponential survival models (15) to assess whether an OAD diagnosis increased the likelihood of a subsequent diagnosis of either CRS or GERD. Piecewise exponential survival models are similar to Cox regression models, but unlike Cox regression models in which the baseline hazard changes with every event, these models allow the baseline hazard to change at a large, but finite number of time points. Because Poisson regression models are mathematically identical to exponential survival models, we used a Poisson likelihood to fit the following models:

(1) $$\log\left\{E\left(Y_{i}|a_{1i},a_{2i},m_{i},c_{i}\right)\right\}=\log\left\{T_{i}\right\}+\alpha_{0}^{*}+\alpha_{1}^{*}a_{1i}+\alpha_{2}^{*}a_{2i}+\alpha_{c}^{*}{\;}^{\prime }c_{i}.$$

(2) $$\log\left\{E\left(Y_{i}|a_{1i},a_{2i},m_{i},c_{i}\right)\right\}=\log\left\{T_{i}\right\}+\alpha_{{\;}_{0}}^{**}+\alpha_{m}^{**}m_{i}+\alpha_{c}^{**}{\;}^{\prime }c_{i}.$$

(3) $$\begin{array}{c} \log\left\{E\left(Y_{i}|a_{1i},a_{2i},m_{i},c_{i}\right)\right\}=\log\left\{T_{i}\right\}+\alpha_{0}+\alpha_{1}a_{1i}+\alpha_{2}a_{2i}+\;\alpha_{m}m_{i}+{\alpha^{\prime }}_{c}c_{i}. \end{array}$$

Here, *Y_i_* is the number of incident cases of the outcome of interest (CRS or GERD) for stratum *i* defined by unique combinations of predictors—age as of September 11, 2001, retirement status (time dependent), last known smoking status (never, former, current), and follow-up time (3-month long-time intervals); *T_i_* is the total follow-up time at risk across all study participants contributing follow-up time to stratum *i*; *a*_1_*_i_* is a dummy variable taking the value of 1 for high exposure and 0 for moderate or low exposure; *a*_2_*_i_* is a dummy variable taking the value of 1 for moderate exposure and 0 for high or low exposure; *m_i_*, the mediator, is a time-dependent variable taking the value 1 for strata including follow-up time after individuals have received OAD diagnoses and 0 otherwise; and *c_i_* is a vector containing all other covariate values for that stratum. In our analyses, we allowed the baseline hazard to change every 3 months, and dummy variables that accomplish this are included in the vector *c_i_*. *Y_i_* is modeled as a Poisson random variable and *m_i_* as a binary random variable, and a full likelihood approach is used. In addition, in piecewise exponential survival models, actual baseline rates can be estimated from the data, and the relative hazards are also relative rates (RRs). Follow-up time began on September 11, 2001, and ended with the earliest of the following: incident GERD or CRS diagnosis date (depending on the outcome of interest), death date, date of latest treatment visit to FDNY-BHS, or the end of the study period (September 10, 2011). Date of latest FDNY treatment visit was used in follow-up time calculation to ensure that participants had an opportunity to receive an aerodigestive diagnosis. We then included diagnoses of OAD as a time-dependent predictor to the models to see how it was associated with CRS and GERD. SAS PROC GENMOD was used to fit the models.

**Figure 1:**
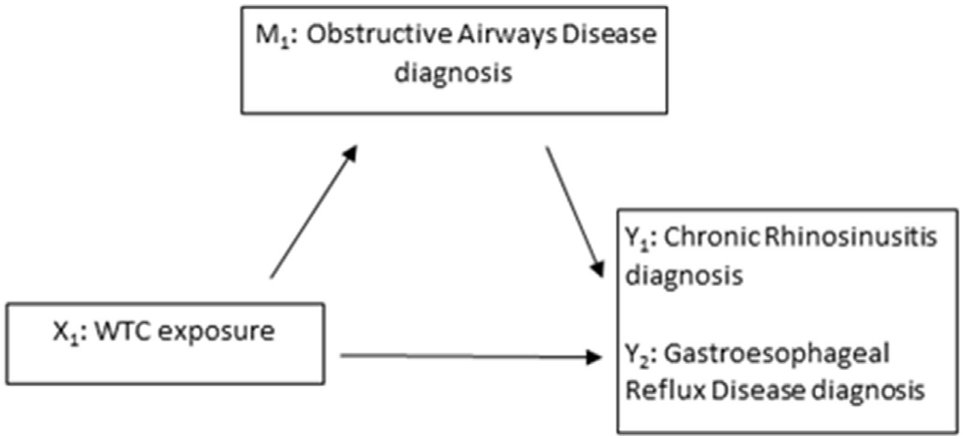
**Model schematic of assessing obstructive airways disease as a mediator (*M*_1_) of the association between World Trade Center (WTC) exposure (*X*_1_) and CRS (*Y*_1_) and between WTC exposure and gastroesophageal reflux disease (*Y*_2_)**.

Mediation, as schematically depicted by Figure 1, is sometimes measured by the difference in effects between a model that includes the mediator such as Eq. 3 and a model that does not include the mediator such as Eq. 1 above. Relevant effects are defined as

(4) $$\begin{array}{l} {\mathrm{RDE}}_{1}=\alpha_{1},\\ {\mathrm{TE}}_{1}=\alpha_{1}^{*}, \end{array}$$

where RDE_1_ = α_1_ is the regression direct effect, the log relative hazard (or RR) of incident CRS or GERD from being in the high- vs. low-exposure group when OAD is included in the model and ${\mathrm{TE}}_{1}=\alpha_{1}^{*}$ is the total effect, the log relative hazard (or RR) of incidence CRS or GERD from being in the high- vs. low-exposure group when OAD is not included in the model. Similar measures are defined for the contrast between moderate- and low-exposure groups. $\alpha_{1}-\alpha_{1}^{*}$ has often been used as a measure of mediation by *m*, but in order for it to be an accurate measure of mediation, the following assumptions must be met: no interaction between the risk factor of interest (here, WTC exposure), no unmeasured confounding, and collapsible measures of effect. The first two assumptions are in doubt in our study, so we also performed a formal causal mediation analysis as follows.

#### Causal Mediation Analysis

To better evaluate the possibility that diagnoses of OAD acted as a mediator for the effect of WTC exposure on the incidence of CRS and GERD, we then fit the following model, similar to that of VanderWeele (16):

(5) $$\begin{array}{c} \log\left\{E\left(Y_{i}|a_{1i},a_{2i},m_{i},c_{i}\right)\right\}=\log\left\{T_{i}\right\}+\theta_{0}+\theta_{1}a_{1i}+\theta_{2}a_{2i}+\theta_{m}m_{i}+\;\theta_{\mathrm{int}1}a_{1i}m_{i}+\theta_{\mathrm{int}2}a_{2i}m_{i}+{\theta^{\prime }}_{c}c_{i}. \end{array}$$

(6) $$\log\left\{\frac{\mathrm{Pr}\left(m_{i}=1|a_{1i},a_{2i},c_{i}\right)}{1-\mathrm{Pr}\left(m_{i}=1|a_{1i},a_{2i},c_{i}\right)}\right\}=\beta_{0}+\beta_{1}a_{1i}+\beta_{2}a_{2i}+{\beta^{\prime }}_{c}c_{i}.$$

Variables are as defined above for Eqs 1–3. SAS PROC HPNLMOD was used for the model fitting. This approach allowed us to directly estimate the following quantities:

(7) $$\begin{array}{l} {\mathrm{CDE}}_{1}=\theta_{1}.\\ {\mathrm{INT}}_{\mathrm{ref}1}=\theta_{\mathrm{int}1}\left(\beta_{0}+{\beta^{\prime }}_{c}c_{i}\right)a_{1i}.\\ {\mathrm{INT}}_{\mathrm{med}1}=\theta_{\mathrm{int}1}\beta_{1}.\\ {\mathrm{PIE}}_{1}=\theta_{M}\beta_{1}.\\ {\mathrm{TE}}_{1}={\mathrm{CDE}}_{1}+{\mathrm{INT}}_{\mathrm{ref}1}+{\mathrm{INT}}_{\mathrm{med}1}+{\mathrm{PIE}}_{1}. \end{array}$$

The controlled direct effect CDE_1_ = θ_1_ is the effect of the risk factor (WTC exposure—being in the high- vs. the low-exposure group) on the outcome (CRS or GERD diagnosis incidence) when the mediator (OAD diagnosis) is not present; ${\mathrm{INT}}_{\mathrm{ref}1}=\theta_{\mathrm{int}1}\left(\beta_{0}+{\beta^{\prime }}_{c}c_{i}\right)a_{1i}$ is the reference interaction between WTC exposure and predictors other than diagnosis of OAD, a measure of the degree of effect modification other than through the mediator; INT_med1_ = θ_int1_β_1_ is the interaction between WTC exposure and the mediator (OAD diagnosis) itself; PIE_1_ = θ*_M_*β_1_ is the pure indirect effect from the product approach; and TE_1_ is the total effect of exposure on incidence. The proportion mediated is PIE_1_/TE_1_ and is of primary interest. As INT_ref1_ depends on the additional model predictors; for our analyses, we used the predictors for the last time interval for a never smoking firefighter aged 40–45 years as of September 11, 2001. Similar quantities were estimated for the effect of being in the moderate- vs. low-exposure group. Approximate confidence intervals were estimated using 1,000 bootstrap replications.

Because the FDNY WTC Health Program started to offer free medications for WTC-related health conditions starting in 2007, we performed sensitivity analyses by refitting all models described above, but beginning follow-up time on September 11, 2006. All analyses were conducted in SAS (SAS v9.4, SAS Institute, Cary, North Carolina).

This study was approved by the Institutional Review Board of the Albert Einstein College of Medicine and Montefiore Medical Center; all study participants gave written consent for research.

## Results

Table 1 shows characteristics of the study population. Most study participants (71%) were in the moderate-exposure group, and over a third (37%) was ever smokers. GERD had the highest post-9/11 incidence (27%), followed by OAD (23%) and CRS (19%). About 96% of those with post-9/11 GERD had at least one endoscopy. Among those with incident OAD, 87% had at least one of the following pulmonary assessments: pulmonary functions testing with bronchodilator response, methacholine challenge tests, or chest CT scans. Among those with incident CRS, 100% had either an abnormal sinus CT or ENT laryngoscopy as at least one abnormal diagnostic test was required in our case definition. There was a clear exposure response gradient for incidences of all three aerodigestive conditions.

**Table 1:** **Characteristics of the Fire Department of the City of New York firefighter study population by World Trade Center (WTC) exposure intensity**.

	High WTC exposure (*N* = 1,473)	Moderate WTC exposure (*N* = 6,391)	Low WTC exposure (*N* = 1,104)	Total (*N* = 8,968)
Age on September 11, 2001 (years), median (IQR)	39.50 (34.60–45.30)	39.30 (34.20–44.90)	40.90 (35.00–46.50)	39.60 (34.40–45.10)
Ever smoker, *N* (%)	546 (37.07)	2,358 (36.90)	442 (40.04)	3,346 (37.31)
Physician visits, median (IQR)	28 (14–47)	28 (14–46)	26 (11–44)	28 (13–46)
Incident gastroesophageal reflux disease (GERD), *N* (%)	462 (31.36)	1,708 (26.73)	254 (23.01)	2,424 (27.03)
Overall obstructive airways disease incidence per 100 person-years	3.56	2.62	1.98	2.68
Overall chronic rhinosinusitis incidence per 100 person-years	2.79	2.23	1.47	2.22
Overall GERD incidence per 100 person-years	3.49	2.92	2.48	2.96

**Figure 2:**
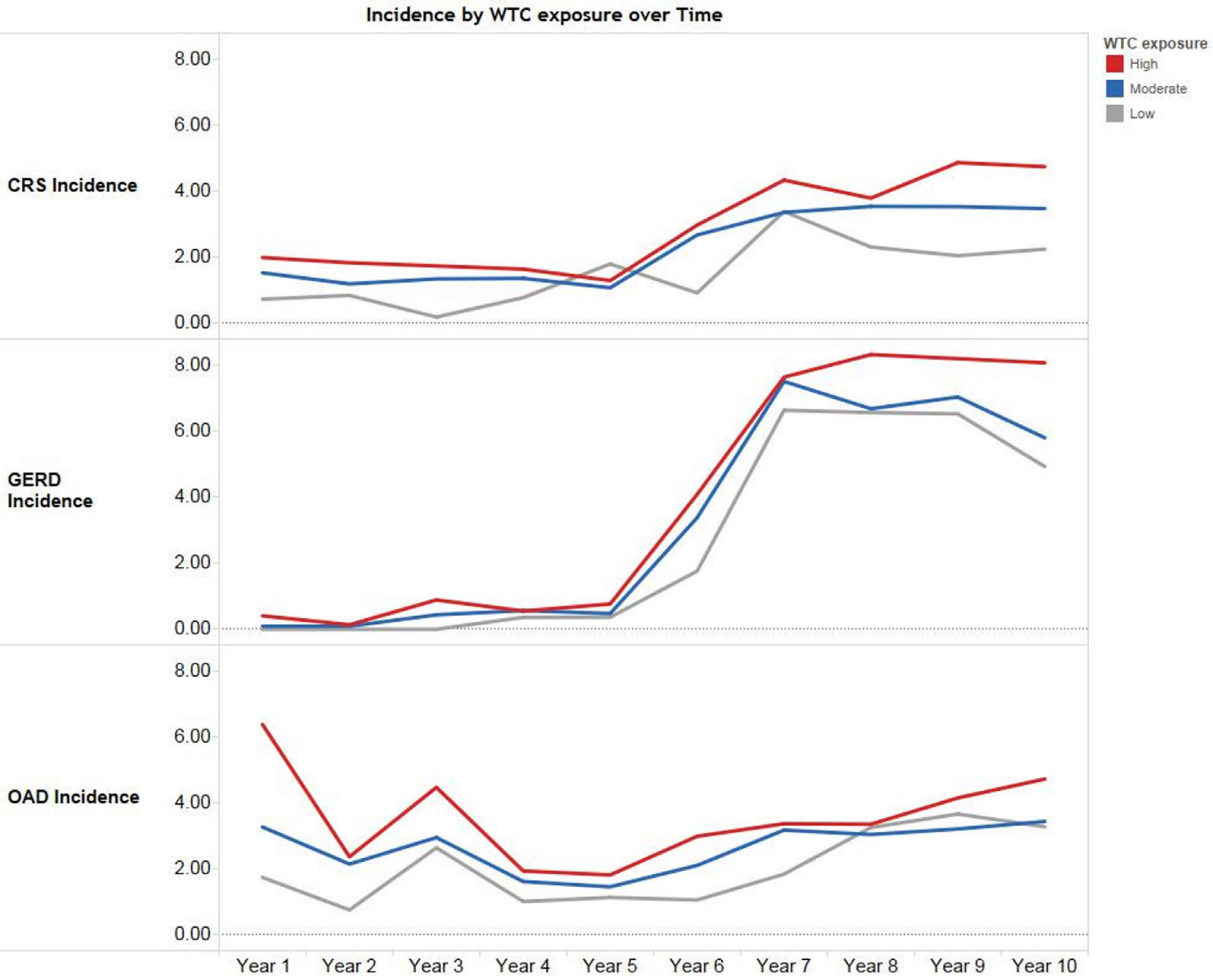
**Crude (unadjusted) incidence rates of aerodigestive conditions by World Trade Center (WTC) exposure intensity over 10 years among World Trade Center-exposed Fire Department of the City of New York (FDNY) firefighters**. Incidence rates are per 100 person-years among 8,968 FDNY firefighters. Years are in 9/11 years, e.g., “Year 1” corresponds to September 11, 2001–September 10, 2002.

Figure 2 shows the crude (unadjusted) incidence rates of OAD, CRS, and GERD by WTC exposure intensity over 10 years of follow-up. Immediately after 9/11, OAD incidence rates were high, whereas the rates of CRS and GERD remained low. Starting in year 6 (2007), rates of all three aerodigestive conditions increased.

In piecewise exponential survival models, individuals with an OAD diagnosis have elevated risks for subsequent diagnoses of either CRS [RR, 4.24 (95% CI, 3.78–4.76)] and/or GERD [RR, 3.21 (95% CI, 2.93–3.52); Table 2]. An OAD diagnosis remains a significant risk factor for both health outcomes after accounting for WTC exposure. Table 2 shows results from Eqs. 1, 2, and 3 above; they suggest a potential role of OAD as a mediator of the association between WTC exposure and CRS and GERD, respectively. Specifically, WTC exposure is significantly associated with both CRS and GERD. After including OAD in each model, the association between WTC exposure and each aerodigestive condition was reduced.

**Table 2:** **Piecewise exponential survival models for the association of WTC exposure, and OAD diagnosis, with CRS and GERD, separately**.

Estimate	Model 1—total effect of WTC exposure OAD ignored		Model 2—effect of OAD diagnosis, WTC exposure ignored		Model 3—regression direct effect of WTC exposure	
	Relative rate	95% CI	Relative rate	95% CI	Relative rate	95% CI
**Models for incidence of chronic rhinosinusitis**						
WTC exposure: high vs. low	1.90	1.56–2.31	N/A		1.70	1.40–2.06
WTC exposure: high vs. moderate	1.28	1.14–1.44	N/A		1.21	1.08–1.36
WTC exposure: moderate vs. low	1.48	1.25–1.76	N/A		1.40	1.18–1.66
Effect of OAD diagnosis	N/A		4.24	3.78–4.76	4.14	3.69–4.65
**Models for incidence of gastroesophageal reflux disease**						
WTC exposure: high vs. low	1.48	1.27–1.73	N/A		1.30	1.11–1.51
WTC exposure: high vs. moderate	1.19	1.07–1.32	N/A		1.12	1.01–1.24
WTC exposure: moderate vs. low	1.25	1.09–1.42	N/A		1.16	1.01–1.32
Effect of OAD diagnosis	N/A		3.21	2.93–3.52	3.16	2.89–3.47

*All models were fit using piecewise exponential survival models and included age, retirement status, smoking status, and season as covariates. Models are defined in the text in Eqs 1–3*.

Table 3 shows the results from the causal mediation analysis, Eqs. 5 and 6 above. The estimates for the controlled direct effect are slightly lower than that of the regression direct effects from the models presented in Table 2: For example, in Eq. 3, the log RR (log hazard ratio) for incidence of CRS in the high- vs. low-exposure group was 0.53 vs. 0.49 in the in the causal mediation model 5 and 6. This was also true for the moderate vs. low exposure contrast (log RRs 0.34 vs. 0.30) and for the corresponding contrasts in the models for GERD incidence (0.26 vs. 0.24 for high vs. low exposure and 0.15 vs. 0.12 for moderate vs. low exposure). Interactions are all small and non-significant, and there is some mediation of the effect of WTC exposure on incidence of CRS and GERD by OAD diagnosis: 10–13% for CRS incidence and 21% for GERD incidence.

**Table 3:** **Results from causal mediation analyses examining the effect of OAD diagnosis as a mediator for chronic rhinosinusitis and gastroesophageal reflux disease**.

	High vs. low exposure			Moderate vs. low exposure		
	Estimate	95% CI		Estimate	95% CI	
**Causal mediation models for chronic rhinosinusitis diagnosis**						
Controlled direct effect	0.485	0.300	0.632	0.302	0.085	0.495
Interaction in absence of mediator	0.011	−0.016	0.042	0.010	−0.007	0.042
Mediated interaction	0.011	−0.013	0.038	0.005	−0.003	0.017
Pure indirect effect	0.061	0.026	0.093	0.028	0.012	0.050
Total effect	0.567	0.377	0.718	0.344	0.137	0.539
Proportion mediated	0.131	0.081	0.190	0.104	0.044	0.208
**Results from conventional models for chronic rhinosinusitis diagnosis**						
Regression direct effect	0.531	0.336	0.723	0.336	0.166	0.507
Total regression effect	0.642	0.445	0.837	0.392	0.223	0.565
**Causal mediation models for gastroesophageal reflex disease diagnosis**						
Controlled direct effect	0.243	0.073	0.427	0.124	−0.024	0.274
Interaction in absence of mediator	0.002	−0.014	0.023	0.003	−0.012	0.023
Mediated interaction	0.002	−0.020	0.025	0.002	−0.008	0.011
Pure indirect effect	0.063	0.036	0.095	0.031	0.015	0.051
Total effect	0.310	0.146	0.486	0.160	0.023	0.302
Proportion mediated	0.211	0.112	0.467	0.206	0.082	0.967
**Results from conventional models for gastroesophageal reflux disease diagnosis**						
Regression direct effect	0.262	0.104	0.412	0.148	0.010	0.278
Total regression effect	0.392	0.239	0.548	0.223	0.086	0.351

*Causal mediation models are defined in Eqs 5 and 6 in the text; conventional models are defined in Eqs 1 and 3. The quantities shown in the tables are defined in Eqs 7 and 4 in the text. Confidence intervals for causal mediation models based on 1000 bootstrap simulations; confidence intervals for conventional models based are large sample Wald-type intervals*.

In sensitivity analyses, we found that the estimates from models in which follow-up started on September 11, 2006, were similar to estimates from models displayed in Table 2 (Table 4). For most contrasts, the proportion mediated was lower than for the complete follow-up (Table 5).

**Table 4:** **Models for incidence of chronic rhinosinusitis and GERD that began follow-up time on September 11, 2006**.

Estimate	Model 1—total effect of WTC exposure, OAD ignored		Model 2—effect of OAD diagnosis, WTC exposure ignored		Model 3—regression direct effect of WTC exposure	
	Relative rate	95% CI	Relative rate	95% CI	Relative rate	95% CI
**Models for incidence of chronic rhinosinusitis**						
WTC exposure: high vs. low	1.93	1.52–2.44	N/A		1.74	1.38–2.20
WTC exposure: high vs. moderate	1.26	1.09–1.46	N/A		1.21	1.04–1.39
WTC exposure: moderate vs. low	1.52	1.24–1.88	N/A		1.45	1.17–1.78
Effect of OAD diagnosis	N/A		3.52	3.07–4.04	3.45	3.00–3.96
**Models for incidence of gastroesophageal reflux disease**						
WTC exposure: high vs. low	1.40	1.20–1.64	N/A		1.25	1.07–1.47
WTC exposure: high vs. moderate	1.16	1.04–1.29	N/A		1.10	0.99–1.23
WTC exposure: moderate vs. low	1.21	1.06–1.39	N/A		1.14	0.99–1.30
Effect of OAD diagnosis	N/A		2.79	2.53–3.07	2.75	2.50–3.03

*All models were fit using piecewise exponential survival models and included age, retirement status, smoking status, and season as covariates. Models are defined in the text in Eqs 1–3*.

**Table 5:** **Results from causal mediation analyses examining the effect of OAD diagnosis as a mediator for chronic rhinosinusitis and gastroesophageal reflux disease for follow-up beginning September 11, 2006**.

	High vs. low exposure			Moderate vs. low exposure		
	Estimate	95% CI		Estimate	95% CI	
**Causal mediation models for chronic rhinosinusitis diagnosis**						
Controlled direct effect	0.542	0.288	0.856	0.352	0.125	0.637
Interaction in absence of mediator	0.001	−0.017	0.013	0.000	−0.007	0.005
Mediated interaction	0.001	−0.018	0.018	0.001	−0.012	0.013
Pure indirect effect	0.029	0.014	0.046	0.015	0.008	0.028
Total effect	0.573	0.333	0.886	0.369	0.148	0.642
Proportion mediated	0.057	0.026	0.108	0.048	0.021	0.101
**Results from conventional models for chronic rhinosinusitis diagnosis**						
Regression direct effect	0.554	0.322	0.788	0.372	0.157	0.577
Total regression effect	0.658	0.419	0.892	0.419	0.215	0.631
**Causal mediation models for gastroesophageal reflux disease**						
Controlled direct effect	0.244	0.069	0.440	0.135	−0.011	0.295
Interaction in absence of mediator	−0.002	−0.016	0.010	0.000	−0.007	0.006
Mediated interaction	−0.002	−0.014	0.012	0.000	−0.011	0.012
Pure indirect effect	0.034	0.018	0.054	0.018	0.008	0.030
Total effect	0.275	0.103	0.464	0.152	0.012	0.305
Proportion mediated	0.136	0.056	0.332	0.289	0.038	0.696
**Results from conventional models for gastroesophageal reflux disease diagnosis**						
Regression direct effect	0.223	0.068	0.385	0.131	−0.010	0.262
Total regression effect	0.336	0.182	0.495	0.191	0.058	0.329

*Causal mediation models are defined in Eqs 5 and 6 in the text; conventional models are defined in Eqs 1 and 3. The quantities shown in the tables are defined in Eqs 7 and 4 in the text. Confidence intervals for causal mediation models based on 1000 bootstrap simulations; confidence intervals for conventional models based are large sample Wald-type intervals*.

## Discussion

We found that WTC-exposed firefighters with an OAD diagnosis had more than four times the risk of subsequently being diagnosed with CRS and three times the risk of being subsequently diagnosed with GERD. Further, we showed that an OAD diagnosis partially mediates the association between WTC exposure and GERD and between WTC exposure and CRS. There are at least two explanations of our findings: biologic, meaning vulnerability of individuals with OAD; and structural, meaning healthcare practices of physicians at FDNY and elsewhere.

In causal mediation analyses, we found that OAD diagnoses mediated 13% of the effect of being in the high vs. low WTC exposure group on the incidence of CRS and 21% of the effect on the incidence of GERD. This is somewhat less than the amount of mediation that would have been estimated by comparing the regression coefficients in models with and without OAD (17 and 34%, respectively). This shows that non-significant interactions, as observed in this study, can affect estimates from the causal mediation analyses. Future studies on mediation should consider the possible effects of interactions on mediation estimates.

Possible biologic explanations include the noteworthy hypothesis that OAD, CRS, and GERD are all a consequence of non-resolving inflammation at aerodigestive mucosal surfaces exposed to caustic WTC dust. Thus, the association of OAD with CRS and with GERD could demonstrate elevated individual risk for mucosal injury due to specific exposure conditions or patient intrinsic vulnerability (3). Another explanation posits that OAD or CRS may cause or exacerbate GERD through mechanically induced inflammation (e.g., cough, postnasal drip, mucous). Third, WTC-exposed individuals with a diagnosis of OAD have diminished capacity for physical activity, possibly leading to reduced fitness and substantial weight gain, which is an independent risk factor for GERD. Finally, OAD medications such as corticosteroids or theophylline may also lead directly to GERD. Non-biologic or structural explanations include the likelihood that individuals with an OAD diagnosis are regularly seen for treatment at FDNY-BHS, which increases the opportunity for those individuals to receive additional diagnoses, including CRS or GERD. At this point, deeper investigation is warranted. The reduced mediation in the sensitivity analyses of the post-2006 follow-up may be evidence for a non-biologic cause early in the follow-up period.

We also acknowledge that reverse causality of the aerodigestive conditions is biologically plausible: that is, CRS or GERD may trigger OAD symptoms such as cough and shortness of breath. If therapeutic interventions intended to improve OAD symptoms were ineffective because the symptoms were due to CRS or GERD, further diagnostic testing could have confirmed CRS or GERD as the etiology of cough and shortness of breath. A main limitation of this study is that the low incidence rates of CRS and GERD in the first 5 years after 9/11 did not allow us to statistically test this hypothesis. Further, the date of diagnosis may not coincide with disease onset if participants delay seeking treatment, which complicates efforts to establish the temporal occurrence, and thus the causal relationship, of OAD, CRS, and GERD. Another limitation is we cannot determine whether individuals with CRS or GERD symptoms sought treatment outside of FDNY-BHS in the immediate years after 9/11, which could have partially accounted for the low incidence rates. Moreover, we observed that incidence rates of CRS, GERD, and OAD all increased after 2007, the year that FDNY WTC Health Program started to provide free medications for WTC-related health conditions. This further complicates our understanding of whether the increased incidence rates after 2007 reflect delayed diagnoses of CRS or GERD or the progressive development of disease. Finally, we acknowledge that there may be unmeasured confounders in this study, which may bias the results of mediation analysis in log-linear models. Although we have included major risk factors for both CRS and GERD as confounders in the respective models, there may be other confounders that we have not captured.

A primary strength of this prospective cohort study is our access to FDNY’s electronic medical records database, which includes dates of diagnoses documented before and after 9/11. We were able to exclude individuals with pre-9/11 evidence of any of three aerodigestive conditions, so we are confident that the diagnoses we included were new post-9/11. Diagnosis dates also allowed us to establish the temporal order of the exposure (WTC-exposure), posited mediator (OAD), and both health outcomes (CRS and GERD), enabling us to conduct mediation analyses.

The combined effects of upper and lower airways disease and GERD have resulted in persistent adverse symptoms, which continue to have a negative impact on the quality of life (11, 12, 17, 18). Study findings support continued medical monitoring of WTC-exposed individuals as well as long-term monitoring and treatment for future disasters with high-intensity exposures.

## Author Contributions

CH conceptualized the study and wrote the first draft of the manuscript with critical revisions from JY, RZ-O, MPW, MW, and DP. XL, JY, RZ-O, JW, and TS contributed to data analysis and data management. All authors read and approved the final manuscript and agree to be accountable for all aspects of the work.

## Conflict of Interest Statement

The authors declare that the research was conducted in the absence of any commercial or financial relationships that could be construed as a potential conflict of interest. The reviewer SDM and handling Editor declared their shared affiliation, and the handling Editor states that the process nevertheless met the standards of a fair and objective review.

## References

[1] Lioy PJ Weisel CP Millette JR Eisenreich S Vallero D Offenberg J Characterization of the dust/smoke aerosol that settled east of the World Trade Center (WTC) in lower Manhattan after the collapse of the WTC 11 September 2001. Environ Health Perspect (2002) 110(7):703–14. 10.1289/ehp.02110703 12117648 PMC1240917

[2] Hall CB Liu X Zeig-Owens R Webber MP Aldrich TK Weakley J The duration of an exposure response gradient between incident obstructive airways disease and work at the World Trade Center site: 2001-2011. PLoS Curr Disasters (2015) 7 10.1371/currents.dis.8a93e7682624698558a76a1fa8c5893f PMC4449208 26064784

[3] Weakley J Hall CB Liu X Zeig-Owens R Webber MP Schwartz T The effect of World Trade Center exposure on the latency of chronic rhinosinusitis diagnoses in New York City firefighters: 2001-2011. Occup Environ Med (2016) 73(4):280–3. 10.1136/oemed-2015-103094 26574577 PMC4819651

[4] Brackbill RM Hadler JL DiGrande L Ekenga CC Farfel MR Friedman S Asthma and posttraumatic stress symptoms 5 to 6 years following exposure to the World Trade Center terrorist attack. JAMA (2009) 302(5):502–16. 10.1001/jama.2009.1121 19654385

[5] Li J Brackbill RM Stellman SD Farfel MR Miller-Archie SA Friedman S Gastroesophageal reflux symptoms and comorbid asthma and posttraumatic stress disorder following the 9/11 terrorist attacks on World Trade Center in New York City. Am J Gastroenterol (2011) 106(11):1933–41. 10.1038/ajg.2011.300 21894225

[6] Kwon S Putman B Weakley J Hall CB Zeig-Owens R Schwartz T Blood eosinophils and World Trade Center exposure predict surgery in chronic rhinosinusitis. A 13.5-year longitudinal study. Ann Am Thorac Soc (2016) 13(8):1253–61. 10.1513/AnnalsATS.201511-742OC 27096198 PMC5021074

[7] Sayuk GS Drossman DA. Gastroesophageal reflux symptoms in 9/11 survivors and workers: insights gained from tragic losses. Am J Gastroenterol (2011) 106(11):1942–5. 10.1038/ajg.2011.357 22056575

[8] Yip J Webber MP Zeig-Owens R Vossbrinck M Singh A Kelly K FDNY and 9/11: clinical services and health outcomes in World Trade Center-exposed firefighters and EMS workers from 2001 to 2016. Am J Ind Med (2016) 59(9):695–708. 10.1002/ajim.22631 27427498

[9] Prezant D Kelly K Jackson B Peterson D Feldman D Baron S In: Centers for Disease Control and Prevention, editor. Use of Respiratory Protection among Responders at the World Trade Center Site – New York City, September 2001. Atlanta: MMWR Morb Mortal Wkly Rep (2002). p. 6–8. 12238539

[10] de la Hoz RE Shohet MR Chasan R Bienenfeld LA Afilaka AA Levin SM Occupational toxicant inhalation injury: the World Trade Center (WTC) experience. Int Arch Occup Environ Health (2008) 81(4):479–85. 10.1007/s00420-007-0240-x 17786467

[11] Wisnivesky JP Teitelbaum SL Todd AC Boffetta P Crane M Crowley L Persistence of multiple illnesses in World Trade Center rescue and recovery workers: a cohort study. Lancet (2011) 378(9794):888–97. 10.1016/s0140-6736(11)61180-x 21890053 PMC9453925

[12] Yip J Zeig-Owens R Webber MP Kablanian A Hall CB Vossbrinck M World Trade Center-related physical and mental health burden among New York City Fire Department emergency medical service workers. Occup Environ Med (2016) 73(1):13–20. 10.1136/oemed-2014-102601 25876606

[13] Prezant DJ Levin S Kelly KJ Aldrich TK. Upper and lower respiratory diseases after occupational and environmental disasters. Mt Sinai J Med (2008) 75(2):89–100. 10.1002/msj.20028 18500710

[14] Prezant DJ Weiden M Banauch GI McGuinness G Rom WN Aldrich TK Cough and bronchial responsiveness in firefighters at the World Trade Center site. N Engl J Med (2002) 347(11):806–15. 10.1056/NEJMoa021300 12226151

[15] Glaser MS Webber MP Zeig-Owens R Weakley J Liu X Ye F Estimating the time interval between exposure to the World Trade Center disaster and incident diagnoses of obstructive airway disease. Am J Epidemiol (2014) 180(3):272–9. 10.1093/aje/kwu137 24980522 PMC4108044

[16] VanderWeele TJ Explanation in Causal Inference: Methods for Mediation and Interaction. USA: Oxford University Press (2015).

[17] Weakley J Webber MP Gustave J Kelly K Cohen HW Hall CB Trends in respiratory diagnoses and symptoms of firefighters exposed to the World Trade Center disaster: 2005-2010. Prev Med (2011) 53(6):364–9. 10.1016/j.ypmed.2011.09.001 21930151

[18] Webber MP Glaser MS Weakley J Soo J Ye F Zeig-Owens R Physician-diagnosed respiratory conditions and mental health symptoms 7-9 years following the World Trade Center disaster. Am J Ind Med (2011) 54(9):661–71. 10.1002/ajim.20993 21966080 PMC3181470

